# Latest Therapeutical Approaches for Triple-Negative Breast Cancer: From Preclinical to Clinical Research

**DOI:** 10.3390/ijms252413518

**Published:** 2024-12-17

**Authors:** Mariona Pont, Marta Marqués, Anabel Sorolla

**Affiliations:** 1 Research Group of Cancer Biomarkers, Biomedical Research Institute of Lleida (IRBLleida), Av. Alcalde Rovira Roure, 80, 25198 Lleida, Spain; mpont@irblleida.cat (M.P.); mmarques@irblleida.cat (M.M.); 2 Department of Medicine, University of Lleida, Av. Alcalde Rovira Roure, 80, 25198 Lleida, Spain

**Keywords:** advanced breast cancer, triple-negative breast cancer, therapy, clinical trial

## Abstract

Triple-negative breast cancer (TNBC) represents roughly one-sixth of all breast cancer patients, but accounts for 30–40% of breast cancer deaths. Due to the lack of typical biomarkers exploited clinically for breast cancer, it remains very difficult to treat. Moreover, its intrinsic high heterogeneity and proneness to become resistant to the drugs administered makes the treatment management very challenging for oncologists. Herein, we outline the different therapies used currently for TNBC and list the ongoing clinical trials to provide an overview of the most recent TNBC therapeutic landscape. In addition, we highlight the emerging therapies in the preclinical stage that hold the most promise, such as epigenetic modulators, CRISPR, miniproteins, radioconjugates, cancer vaccines, and PROTACs. Moreover, we navigate through the existing limitations and challenges which hamper the development of new and more effective treatments for TNBC. Lastly, we point to emerging new directions that may revolutionize future therapy for TNBC.

## 1. Introduction

### 1.1. Breast Cancer

Breast cancer was the second most diagnosed cancer worldwide and the first cause of cancer-related death in females in 2022 [1]. Breast cancer is a highly heterogenous neoplasm which is typically classified immunohistochemically for the presence or absence of estrogen receptor (ER), progesterone receptor (PR), and human epidermal growth factor receptor 2 (HER2). According to this classification, there are ER+, HER2+, and TNBC (ER-, PR-, and HER2-) breast cancer tumors. The molecular classification of breast cancer consists of the luminal A and B subtypes, which overlap with ER+ breast cancer; the HER2-enriched subtype, which overlaps with HER2+ breast cancer and can be *TP53*-mutated or wild type; and basal-like breast cancer, which overlaps with TNBC, and the claudin-low breast cancer subtype [2]. Breast cancer subtypes vary in their biology and behavior, with TNBC being the one having the highest proliferation rates, alterations in DNA repair genes, and genomic instability, as well as being the most aggressive, with a higher risk of early relapse and metastasis and a higher mortality rate [3]. Notably, 50% of early-stage (I–III) TNBC patients experience recurrence, and 37% of patients die within the first five years after surgery, which portends a very poor prognosis [4]. This is specially desolating given that TNBC accounts for 15–20% of all breast carcinomas and more frequently affects younger premenopausal women under the age of 40 [5].

### 1.2. TNBC Characteristics

**Figure 1:**
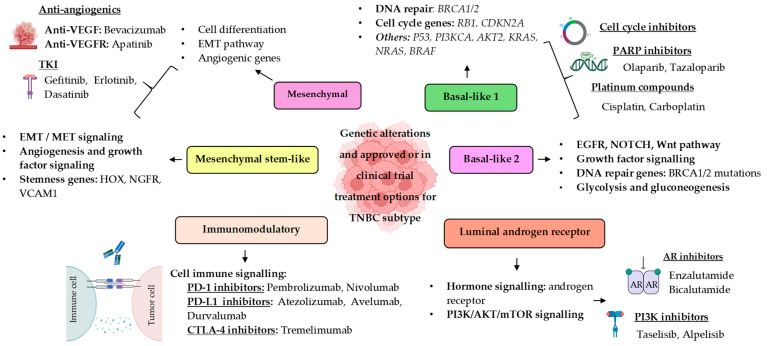
Schematic representation of Lehmann’s TNBC molecular subtypes, their genetic alterations, and therapeutic approaches.

According to Lehmann et al. [6], TNBC can be classified into six molecular subtypes: the basal-like 1 (BL1), basal-like 2 (BL2), mesenchymal (M), mesenchymal stem-like (MSL), immunomodulatory (IM), and luminal androgen receptor (LAR) subtypes (Figure 1). The BL1 subtype is characterized by the upregulation of genes related to the cell cycle and DNA damage response, while BL2 is distinguished by an enrichment of growth factor signaling, glycolysis, and gluconeogenesis pathways. The mesenchymal (M) and mesenchymal stem-like (MSL) subtypes show high expression of genes associated with motility and epithelial–mesenchymal transition (EMT). In contrast, the MSL subtype exhibits a decreased expression of proliferation-related genes but is enriched in genes related to mesenchymal stem cells. The immunomodulatory subtype (IM) is characterized by the abundant expression of genes involved with immune cell processes such as antigen presentation and immune signaling transduction pathways. Finally, the luminal androgen receptor (LAR) subtype expresses genes involved in steroid synthesis, androgen/estrogen metabolism, and porphyrin metabolism. These subtypes reflect differences in signaling pathways, genetic and epigenetic characteristics, and the composition of the immune microenvironment, resulting in distinct clinical outcomes and responses to treatment [7].

Some authors have divided TNBC into AR-positive and AR-negative TNBC based on the expression of the androgen receptor (AR). AR-negative TNBC is also known as quadruple-negative breast cancer (QNBC). There exist differences regarding the tumor biology and molecular profiles between QNBC and TNBC. For instance, QNBC has higher expression of epidermal growth factor (EGF) and genes involved in immune response, which results in higher proliferative and immunogenic properties when compared to TNBC, leading to a worse prognosis. For this reason, a distinct subtype classification for QNBC, independent of TNBC, is recommended [8,9].

### 1.3. Genetic and Epigenetic Alterations Commonly Found in TNBC

Continuing with Lehmann’s classification, different researchers have performed targeted and whole-exome sequencing to further define the genetic characteristics of the different subtypes. Bareche et al. [10] reported the genomic alterations of each TNBC subtype. BL1 tumors are characterized by having high levels of chromosomal instability; high rates of *TP53* loss mutations; copy number gains; and amplifications in *PI3KCA*, *AKT2*, *KRAS*, *NRAS,* and *BRAF*. This subtype also shows deletions in genes involved in DNA repair mechanisms such as *Breast cancer gene 1/2* (*BRCA1/2*) and *Retinoblastoma protein 1* (*RB1*), in addition to genetic gains in *PPAR1*. All this suggests that BL1 tumors may respond well to Polyadenosine 5′-diphosphoribose polymerase (PARP) inhibitors, specific kinase inhibitors, and Phosphoinositide 3-kinase/Protein kinase B (PI3K/AKT) inhibitors. The LAR subtype shows mutations in *PI3KCA*, *AKT1*, and *CDH1* genes, being a good candidate for PI3K and AKT inhibitors. Additionally, this subtype shows higher expression of cyclin-dependent kinase 4/6 (CDK4/6) suggesting they might be suitable for CDK4/6 inhibitors. The MSL and M subtypes are associated with an overexpression of genes related to angiogenesis, making them potential candidates for angiogenic therapies. The M subtype shows increased activity in the epidermal growth factor receptor (EGFR) and NOTCH signaling pathways, while the IM subtype demonstrates high expression levels of immune response genes and checkpoint inhibitor genes including cytotoxic T-lymphocyte-associated antigen-4 (CTL4), programmed cell death protein-1 (PD-1), and PD-ligand 1 (PD-L1) [11].

Another group has stratified TNBC into different subtypes on the basis of intrinsic oncogenic alterations, underscoring the heterogeneity of the disease and uncovering therapeutic targets [12].

In general, for all subtypes, the most prevalent genetic alterations found are in *TP53* and *PI3KCA*, followed by *BRCA1*, *RB1*, *Ataxia-telangiectasia mutated* (*ATM*), and *Phosphatase and tensin homolog* (*PTEN*). Genes affected by somatic copy number alterations (CNAs) include *EGFR*, *PTEN*, *CCND1*, *RB1,* and *CCNE1*, with *MYC* amplification being the most frequent CNA in TNBC [13,14].

Epigenetic dysregulation as well as genetic alterations are well-recognized hallmarks of cancer, given its association with cell cycle control and proliferation [15]. The most studied epigenetic aberrations involve changes in DNA methylation patterns, histone modification, and some protein regulators [16]. Hypermethylation of tumor suppressor gene promoters, which is related to gene silencing, hypomethylation across the genome, and the disbalance of histone methyltransferases and deacetylases, is a key regulator of cancer progression, metastasis, and immune evasion [17]. Epigenetic alterations also have a role in TNBC progression. Regarding DNA methylation, the numbers of CpG are similar between TNBC and non-TNBC, but TNBC exhibits distinct genes that are affected, such as the hypermethylation of *Cyclin-dependent kinase inhibitor 2B* (*CDKN2B*) and the non-methylation of *Glutathione S-transferase pi 1* (*GSTP1*), among others. Zhu et al. reported that hypermethylation in the *BRCA1* gene is present in over 50% of TNBC patients, with a two-fold higher frequency in basal-like TNBC compared to non-basal-like subtypes [18]. The methylation profile of TNBC tumors has been defined by the methylation of five genes (*CDKN2B*, *CD44*, *MGMT*, *RB1*, and *p73*) plus the non-methylation of 11 genes (*GSTP1*, *PMS2*, *MSH2*, *MLH1*, *MSH3*, *MSH6*, *DLC1*, *CACNA1A*, *CACNA1G*, *Twist1*, and *ID4*) [19]. Histone modifications also play a role in TNBC affecting the extracellular matrix components. Higher levels of lysine acetylation and methylation (H3K4ac and H3K4me3) in TNBC cell lines have been observed to promote metastasis and invasion. Moreover, several miRNAs have been associated with disease progression, EMT, extracellular matrix composition, invasiveness, migration, etc. For example, it has been observed that the miR-9 family is upregulated in TNBC cell lines, leading to activation of the β-catenin pathway and upregulation of vascular endothelial growth factor (VEGF). In contrast, the miR-200 family is downregulated, demonstrating a tumor-suppressive role. Finally, several long non-coding RNAs have also been associated with different extracellular matrix components, such as *HAS2-AS1*, *HOTAIR,* and *LINK-A* [19].

## 2. Current Treatments Approved for the Treatment of TNBC

Since TNBC lack the expression of ER and PR and have amplification or overexpression of HER2, hormonal therapies designed to inhibit ER and PR and anti-HER2 treatment are not effective. Moreover, the high heterogeneity of TNBC tumors makes it very difficult to find a universally useful targeted therapy. Indeed, there is no available biologically effective targeted therapy for TNBC yet. Current therapeutic strategies for advanced TNBC include chemotherapy, immunotherapy, drug-based targeted therapies, and antibody–drug conjugates (ADCs). Note that the terms advanced and metastatic breast cancer are not completely synonymous. Advanced is a term frequently used by the physician that refers to the likelihood that a breast cancer can be controlled or cured, with “advanced” meaning very unlikely to be cured. Metastatic breast cancer means that the breast cancer has spread to distant organs or body sites. Regarding the therapy implications for metastatic disease, it is important to point out that metastatic TNBC is lethal, and the general assumption is that metastases share the same oncogenic alterations as primary lesions. However, there is evidence for overlapping yet distinct oncogenic alterations that drive metastasis versus primary lesions through clonal expansion and parallel evolution. The administration of the therapy would depend on the molecular characteristics of the tumors, the disease’s stage, and the patient’s age [20].

### 2.1. Chemotherapy

Systemic chemotherapy is the mainstay treatment for TNBC. The type of drug used depends on the stage and the mutational landscape of these cancers. For stages I–III TNBC, chemotherapy or the PD-1 inhibitor Pembrolizumab is preferred in the neoadjuvant setting. If cancer tissue remains after surgery, the patient is administered Capecitabine, Pembrolizumab, or the PARP enzyme inhibitors Olaparib or Talazoparib. The administration of Olaparib is specially indicated in those tumors harboring *BRCA* mutations. For stage IV, the chemo drugs of choice are anthracyclines (e.g., Doxorubicin and Epirubicin), taxanes (Docetaxel, Paclitaxel and Cabazitaxel), Capecitabine, Gemcitabine, Eribulin, etc., either alone or in combination. Nab-paclitaxel (albumin-bound Paclitaxel) is preferred over Paclitaxel as it has been shown to improve the toxicity profile of Paclitaxel [21,22].

### 2.2. Immunotherapy

PD-L1, PD-1, and Cytotoxic T-Lymphocyte Antigen 4 (CTLA-4) are the main immune-related proteins that participate in the design of blocking antibodies for immunotherapy. The corresponding agents have also been tested for efficacy in TNBC. Pembrolizumab and Nivolumab are monoclonal antibodies targeting PD-1 that received approval a decade ago from the U.S. Food and Drug Administration (FDA) for treating multiple solid tumors. Regarding TNBC, in 2016, Muro et al. investigated the safety of Pembrolizumab in previously treated patients with advanced TNBC in a phase I clinical trial (KEYNOTE-012, NCT01848834), finding evidence of clinical benefit and acceptable safety [23]. Another phase II study (KEYNOTE-086, NCT02447003) in 2019 evaluated Pembrolizumab as a first-line therapy for PD-L1-positive metastatic (m)TNBC patients, demonstrating a favorable safety profile and antitumor activity [24]. Both trials reported that Pembrolizumab had a durable and safe antitumor effect. In 2020, the phase III clinical trial KEYNOTE-522 (NCT03036488) revealed the superior efficacy of combining Pembrolizumab with chemotherapy in the neoadjuvant setting compared to chemotherapy alone in terms of complete response rate and event-free survival [25]. Based on this evidence, the FDA granted the approval of Pembrolizumab for high-risk, early-stage TNBC. Later, a clinical trial comparing Pembrolizumab against chemotherapy for mTNBC (KEYNOTE-119-NCT02555657) showed that, while Pembrolizumab alone did not significantly improve overall survival (OS), further research may be needed, as the authors suggested that this drug may have benefits for specific subsets of patients with PD-L1-enriched tumors [26]. The combination of Pembrolizumab with other drugs has also been investigated. Pembrolizumab combined with chemotherapy (Carboplatin and Gemcitabine, Paclitaxel) has been studied in patients with previously untreated locally recurrent inoperable or metastatic TNBC, resulting in significantly longer OS than chemotherapy alone (KEYNOTE-355, NCT02755272) [27]. Furthermore, Pembrolizumab has been combined with Eribulin, a microtubule inhibitor, showing good tolerance and antitumor activity in mTNBC (KEYNOTE-150, NCT02513472) [28].

PD-L1 inhibitors Atezolizumab, Avelumab, and Durvalumab have been studied to treat TNBC in different clinical trials. Atezolizumab demonstrated a safe and tolerable profile and clinical benefit as monotherapy (NCT01375842) in combination with Nab-Paclitaxel in metastatic (m)TNBC patients previously treated with chemotherapy (NCT01633970) and in untreated mTNBC patients. It also led to prolonged progression-free survival (IMpassion130-NCT022425891) [29,30]. Another clinical trial (NeoTRIPaPDL1) found that Atezolizumab with Carboplatin and Paclitaxel increased pathological response in stage II/III TNBC. Moreover, Avelumab showed a safe, well-tolerated, and beneficial profile in the JAVELIN trial for PD-L1 positive mTNBC. Lastly, Durvalumab has been tested with a tumor vaccine to increase the activation of T-cells and promote PD-L1 expression to enhance the effect of the drug (NCT02725489). Other studies have explored the combination of Durvalumab with Paclitaxel (NCT02628132); Olaparib, a PARP inhibitor; or Cediranib, a VEGFR inhibitor (NCT02484404).

Finally, regarding CTLA-4 inhibitors, Tremelimumab has been investigated in advanced solid tumors, including TNBC in a phase II trial (NCT02527434), and has been studied in a phase I trial combined with radiotherapy, demonstrating good tolerability, but further research is needed to optimize this treatment.

A key challenge in the field of immunotherapy is the identification of predictive biomarkers to effectively determine which patients are most likely to benefit from these treatments, as the presence of PD-L1 in the tumor does not always predict a good response to immunotherapy. In this sense, in addition to PD-L1 expression, factors such as tumor mutational burden (TMB) and tumor-infiltrating lymphocytes (TILs) have been explored as promising biomarkers [31].

### 2.3. Targeted Therapy

#### 2.3.1. PARP Inhibitors

TNBC harboring *BRCA1* or *BRCA2* mutations is especially indicated for treatment with PARP inhibitors (PARPis). Different PARPis are approved for the treatment of some BRCA-associated cancers: ovarian, pancreatic, prostate, and breast cancer [32]. In breast cancer, including TNBC, Olaparib and Talazoparib were approved in 2018 for the treatment of germline-mutated *BRCA1*/2, also known as gBRCAm, metastatic HER2-negative breast cancer. Olaparib was compared with standard chemotherapy agents such as Capecitabine, Vinorelbine, or Eribulin in the Phase III OlympiAD Trial (NCT02000622), showing a reduced risk of disease progression or death with Olaparib. Additionally, the median progression-free survival (PFS) was longer with Olaparib than with standard treatments [33]. Similarly, results from the EMBRACA trial (NCT01945775) demonstrated that Talazoparib offers significant advantages over conventional chemotherapy, such as Capecitabine, Vinorelbine, Eribulin, or Gemcitabine, in PFS. However, none of these PARPis have led to significant improvement in OS compared to standard treatments. Nevertheless, both have exhibited a lower incidence of severe adverse events and improved PFS [34]. In addition to Olaparib and Talazoparib, other PARPis are currently under investigation in TNBC, such as Rucaparib, Niraparib, and Veliparib. Other clinical trials are investigating PARPi in combination with other therapies, such as immunotherapy, with the immune-checkpoint inhibitors Pembrolizumab or Durvalumab. In two different phase II trials, the combination of Niraparib with Pembrolizumab (TOPACIO-NCT02657889) or Olaparib with Durvalumab (MEDIOLA-NCT02734004) showed promising results in terms of safety, tolerability, and overall response in patients with gBRCAm metastatic TNBC. Other studied combinations include PARP inhibitors with other targeted therapies, such as ATM and PI3KCA inhibitors [35].

#### 2.3.2. Androgen Receptor Antagonists

One of Lehmann’s subtypes is the LAR subtype. This subtype is characterized by the expression of AR, a steroid hormonal receptor that regulates various genes involved in cellular processes such as proliferation or apoptosis, and in TNBC carcinogenesis. Due to the positive response of AR inhibition in prostate cancer to drugs like Bicalutamide, Enzalutamide, or Apalutamide, it has been considered that TNBC could also benefit from them. Indeed, the phase II study (NCT01889238) where researchers investigated the administration of Enzalutamide in advanced AR-positive TNBC demonstrated a clinical benefit and good tolerability with a safety profile of the drug, supporting it as a treatment for advanced TNBC [36].

Combination treatments with AR inhibitors have been also explored. The PI3K/AKT/mTOR signaling pathway is often activated in TNBC, and is especially present in the LAR subtype. This suggests that combining AR inhibitors with PI3K inhibitors would have a synergistic effect and provide a benefit to these patients. Some clinical trials have investigated the efficacy and safety of this combination. Lehmann et al. showed that the combination of Enzalutamide with Taselisib increased the clinical benefit rate in TNBC patients with AR+ tumors (NCT02457910) [37]. Other clinical trials have studied the efficacy and safety of Alpelisib combined with Enzalutamide in patients with AR+ and PTEN+ metastatic breast cancer, including TNBC (NCT03207529). Luo et al. demonstrated a positive correlation among AR, PARP1, and BRCA1 expression in TNBC, suggesting that the combination of AR and PARP inhibitors could be a strategy for TNBC. They demonstrated that combining Bicalutamide and PARP inhibitor ABT-888 could inhibit cell viability and induce cell apoptosis in vitro or in vivo in AR-positive TNBC [38].

#### 2.3.3. EGFR Inhibitors

EGFR is involved in angiogenesis, cell proliferation, metastasis, and inhibition of apoptosis, and it is commonly overexpressed in various cancers, including TNBC. Several anti-EGFR therapies are used clinically, including tyrosine kinase inhibitors like Gefitinib and Erlotinib for cancers like non-small-cell lung cancer (NSCLC) and pancreatic cancer. Other monoclonal antibodies like Cetuximab, Panitumumab, and Necitumumab are used to treat cancers such as colorectal cancer, head and neck squamous cell carcinoma, and squamous cell lung cancer [39]. Among all breast cancer subtypes, EGFR is more frequently overexpressed in TNBC, and its high expression is associated with poor prognosis. This fact makes EGFR inhibitors an attractive option for TNBC treatment. Some clinical trials testing EGFR inhibitors, such as Gefitinib, Erlotinib, and Afatinib, have shown minimal or no response in mTNBC. Other studies have demonstrated that the use of anti-EGFR monoclonal antibodies, such as Cetuximab or Panitumumab, has a slightly better effect than EGFR inhibitors, but the efficacy is limited. Several clinical trials are examining the possibility of combining anti-EGFR monoclonal antibodies with chemotherapy to improve the efficacy [39].

#### 2.3.4. Vascular Endothelial Growth Factor Inhibitors

VEGF and its receptor VEGFR are involved in angiogenesis, stimulating cellular pathways that promote the formation of blood vessels, leading to rapid tumor growth and metastasis. This fact makes anti-angiogenic therapies targeting VEGF or VEGFR a viable treatment strategy for TNBC.

Bevacizumab is an antibody targeting VEGF whose effect has been studied in combination with Paclitaxel and Capecitabine, demonstrating a high antitumor activity and safety profile in TNBC patients [40] (GINECO A-TaXel phase II study). Moreover, the combination of Bevacizumab with Carboplatin and Paclitaxel in a phase II clinical trial (NCT03577743) showed an improvement of PFS and response rate in mTNBC patients. Additionally, some phase III trials (E2100, AVADO, RIBBON-1, and RIBBON2) have shown an enhancement in the overall response rate and/or PFS and a safety profile by combining Bevacizumab with chemotherapy (Capecitabine, Docetaxel, Gemcitabine, Vinorelbine and Paclitaxel) for first-line or second-line therapy in patients with mTNBC [41,42].

In the neoadjuvant setting, various studies have combined Bevacizumab with chemotherapy; however, the results were not conclusive and could not be replicated in other trials. Additional trials must be performed to identify which patients can benefit and which biomarkers can be used to predict the response and select the convenient treatment [42].

Besides VEGF inhibitors, there are also drugs that target VEGFR, such as Apatinib. In a phase II clinical trial (NCT03394287), the researchers demonstrated that Apatinib combined with Camrelizumab, an anti-PD1 antibody, had a favorable clinical effect and safety profile in patients with advanced TNBC [43]. Other clinical trials combined Apatinib and Camrelizumab with Eribulin (NCT04303741) [44] or with Fuzuloparib, a PARP inhibitor (NCT03945604), demonstrating, in both triple combinations, a safety profile and antitumor effect in advanced TNBC patients [45].

### 2.4. Antibody–Drug Conjugates

Antibody–drug conjugates (ADCs) deserve a special mention due to the implications that they have had for TNBC treatment. ADCs represent a new class of therapies designed to deliver chemotherapeutic agents directly to tumors, using the affinity between antibodies and specific antigens that are overexpressed on cancer cells. The structure of ADCs consists of three main components: a monoclonal antibody that targets the antigen on the cancer cell, a potent cytotoxic agent that induces cell death upon release, and a linker that connects the antibody and the cytotoxic agent.

ADC is currently a viable alternative for advanced/metastatic TNBC in patients who have been previously treated with at least two different options. Currently, three ADCs are available for the treatment of breast cancer: Sacituzumab govitecan (SG), Trastuzumab deruxtecan, and Trastuzumab emtansine. Trastuzumab refers to an anti-HER2 monoclonal antibody. SG is the only ADC approved for TNBC in 2023, indicated for patients with unresectable locally advanced or metastatic TNBC who have received at least two prior systemic treatments, one of which must have been for advanced disease. Trastuzumab deruxtecan is indicated for patients with advanced HER2-positive breast cancer after one or more prior anti-HER2 therapies. Lastly, Trastuzumab emtansine is approved for HER2-positive breast cancer and is used as a post-neoadjuvant therapy in early-stage HER-2-positive BC with residual disease following neoadjuvant treatment with Trastuzumab and taxanes [46].

The mode of action of SG is targeting the trophoblast cell surface antigen 2 (Trop2), a protein that is highly overexpressed in triple-negative breast cancer (TNBC) and associated with poor prognosis. Once there, SG delivers SN-38, the active metabolite of topoisomerase I inhibitor irinotecan, inducing cancer cell death. While approved as a monotherapy, other studies are exploring its combination with other treatments. Bardia et al. [47] showed in a phase I study that combining SG with Talazoparib, a PARP inhibitor, may stop cancer growth and disrupt DNA repair pathways (NCT04039230). Additional studies suggest that combining SG with platinum compounds or anti-apoptosis protein blockers may enhance its efficacy. Nevertheless, it remains necessary to investigate the effects of prolonged exposure to these combinations to determine if acquired resistance occurs and, if so, which mechanisms are responsible for the acquisition of resistance [48].

## 3. Currently Ongoing Clinical Trials for TNBC Treatment

In this section of the review article, we focus on the ongoing recruiting clinical trials in advanced or mTNBC listed in ClinicalTrials.gov., which are summarized in Table 1. On 21 October 2024, there were 42 clinical trials of this type. Within them, the authors investigated different therapeutic approaches, including immunotherapy, antiangiogenic drugs, antibody–drug conjugates, and bispecific antibodies, either alone or in combination with chemotherapy such as Eribulin, Capecitabine, Gemcitabine, or Vinorelbine. Their primary goal was mostly to assess the overall response rate, PFS, and OS. Other endpoints were the assessment of the duration of the response; the type, quantity, and severity of adverse effects; and the evaluation of health-related quality of life, enabling researchers to evaluate the clinical impact and the well-being of the patients during the treatment.

## 4. Emerging Therapies and Preclinical Approaches for the Treatment of TNBC

### 4.1. Epigenetic Modulators

The reversibility of epigenetic modifications represents a very attractive therapeutic approach in cancer. Among the most common mechanisms of modulation of the epigenome, the inhibition of DNA methyltransferases (DNMTi), overexpression or activation of DNA methyltransferases (DNMT), inhibition of histone-modifying enzymes (e.g., histone deacetylase inhibitors, HDACi), overexpression or activation of histone-modifying enzymes, inhibition or activation of DNA methyltransferases or histone-modifying enzymes by substrate depletion, targeted removal or addition of methyl groups, and targeted modification of histone proteins [49] can be highlighted. The mode of action of “epidrugs” can be through cellular delivery, like small molecules, or via genetic engineering. However, the main disadvantage of epigenetic drugs as therapeutic agents is the lack of specificity, as all cell types are targeted, and when focusing on one single cell, countless genomic loci are also targeted. A phase II clinical trial combining the DNMTi 5-Azacitidine (5-AZA) and the HDACi Entinostat in TNBC and hormone-resistant breast cancer patients demonstrated that epigenetic therapy alone only provides a partial response [50]. Consequently, the combination of Entinostat and immunotherapy was used in histologically or cytologically confirmed mTNBC in an ongoing study at the Center for Cancer Research at the National Cancer Institute (NCT04296942) [51].

### 4.2. Clustered Regularly Interspaced Short Palindromic Repeats Technology

Clustered Regularly Interspaced Short Palindromic Repeats (CRISPR) technology has many applications in TNBC, from modeling the disease to studying the pathogenesis of the disease, going through diagnosis and therapy [15]. At the time when we wrote this section of the review article (12 November 2024), there were five clinical trials listed using CRISPR/Cas9 in solid tumors (Clinical.Trials.gov). Of them, one was completed in advanced breast cancer patients (NCT05812326). However, no results have been released so far. There are very few studies published in the literature using Chimeric Antigen Receptor (CAR) T-cells engineered in vitro with CRISPR/Cas9 in solid tumors, which is, so far, the only type of therapeutic approach investigated in both solid tumors and hematologic malignancies. One example is the utilization of CD70-targeted allogenic CAR T-cell therapy in 16 patients with relapsed/refractory renal cell carcinoma, demonstrating disease control in over 80% of the patients and one durable complete response [52]. Another published example is non-virally knocking down the genes TRAC and TRBC encoding for T-cell receptors (TCRs) and knocking in neoantigen-specific TCR in 16 patients, including refractory melanoma, breast cancer, ovarian cancer, and colorectal cancer patients. This study was a proof of concept of genome editing and cloning in vitro and a dose-scalation study where five patients achieved stable disease [53]. There are no ongoing clinical trials regarding the utilization of CRISPR/Cas9 as a direct treatment for TNBC. However, the design of new clinical trials via T-cell therapy is anticipated in the near future.

### 4.3. Miniproteins

Miniproteins or peptides, in the form of shorter versions of native proteins, act as dominant-negative agents, interfering in protein–protein interactions between two partner proteins, blocking their action and the associated downstream processes. Miniproteins have been used to inhibit certain oncotargets in the context of TNBC. The excellent cell penetration properties of miniproteins make them ideal candidates for targeting intracellular proteins. Moreover, they are able to bind to featureless interfaces of transcription factors, which are beyond the reach of classical therapeutics [54]. Our group has designed various miniproteins able to bind and block protein–protein interactions characteristic of the transcription factors ENGRAILED, SOX2, and MYC in TNBC [55,56,57]. Other groups have investigated other miniproteins in TNBC. This is the case of KJ-Pyr-9 against MYC in the TNBC cell line MDA-MB-231 [58], an inhibitory peptide against HOX [59] in MDA-MB-231 cells or Omomyc against MYC in MDA-MB-231 cells and patient-derived TNBC xenografts [60]. So far, Omomyc is the only miniprotein-based inhibitor that has reached the clinical setting [61]. In this phase I clinical trial examining tolerability and safety as primary outcomes in 21 patients, a TNBC patient was included. Omomyc caused mild side effects in the patients studied [61]. The second part of this study includes 18 TNBC patients, and the results are still to be published. Despite the great potential of miniproteins, they show some limitations, such as a short-half life in circulation and immunogenicity. The short half-life due to enzymatic degradation by proteases and fast renal clearance reduces their bioavailability. Strategies to overcome this limitation include the introduction of levorotatory (L) amino acids, unnatural amino acids, and chemical modifications in short linear peptides such as cyclization [54]. In particular, synthetic cyclic peptides could serve as targeting ligands for therapeutics used in TNBC. An example is cyclic peptides recognizing and binding with high affinity to the tumor integrin α_v_β_6_ [62], overexpressed in TNBC [63].

### 4.4. Radioconjugates

Radioconjugates are emerging theranostic molecules being investigated for both cancer diagnosis and treatment. They consist of a molecule with high affinity for a particular protein in the cancer cell and a radionuclide, which is able to locally deliver radiation energy to kill the cancer cells while exerting minimal damage to the surrounding tissue. There have only been a few studies utilizing radioconjugates preclinically in TNBC. Hernandez et al. synthesized and tested 177Lu-NM600, emitting β-particles and consisting of an alkylphosphocholine named NM600 labeled with 177Lu in 4T1 allografts. They observed a complete response of tumor growth reduction in 60% of the mice analyzed with minimal side effects [64]. Heesch et al. utilized 177Lu conjugated with a peptide with affinity for prostate-specific membrane antigen (PMSA), emitting β-particles, in BT-20, Hs578T, and MDA-MB-231 cells. Such a radioligand, namely, [177Lu]Lu-PSMA, induced apoptosis in the associated endothelial cells [65]. A very similar radioconjugate, 177Lu-PSMA-617 or Lutetium Lu 177 vipivotide tetraxetan or Pluvicto, was approved by the FDA in 2022 for the treatment of PSMA-positive metastatic castration-resistant prostate cancer treated with AR pathway inhibition and taxane-based chemotherapy after observing an extension of OS [66]. Finally, Radaram et al. investigated the effects of the radioconjugate 89Zr emitting γ radiation conjugated to an anti-PD-L1 antibody in BT-549 cells and xenografts, demonstrating effective PD-L1 binding and biodistribution [67].

### 4.5. Cancer Vaccines

An interesting treatment strategy for TNBC is cancer vaccines. Different vaccines have been produced to target antigens on cancer cells or cancer stem cells (CSC) [68]. Within cancer cell antigens, we can highlight folate receptor α (FRα), α-Lactoalbumin, HER2, HER3, Carcinoembryonic antigen (CEA), Brachyury, Mucin-1 (MUC1), X-box binding protein 1 (XBP1), CD138, CS-1, Survivin and Topoisomerase 2 alpha (TOP2A). Regarding the CSC antigens investigated, they are CD105, Y box-binding protein 1 (YBX1), SRY (sex determining region Y)-box 2 (SOX2), Cadherin 3, and Mouse double minute 2 homolog (MDM2). The vaccines produced for TNBC have different methods of immunization. They can be from mRNA, DNA, whole protein, viral vectors, or peptides [69]. The administration of vaccines has been performed alone and in combination with chemotherapeutic drugs or immunotherapy. When in combination, the therapeutic agents anti-PD-L1 Durvalumab and Avelumab, the anti-PD-1 Sasanlimab and Pembrolizumab, the anti-CTLA-4 Tremelimumab, Cyclophosphamide, and Capecitabine are the most used ones [69]. Newer antigens for TNBC vaccines are currently being identified using more complex techniques than the traditional SEREX, such as RNA sequencing, human leukocyte antigen (HLA) typing, and machine learning methods. Currently, there are 34 listed clinical trials investigating vaccines in TNBC (ClinicalTrials.gov). Of them, there is one study in phase III evaluating the efficacy and safety of the anti-Globo H vaccine Adagloxad simolenin (OBI-822)/OBI-821 in Globo-H positive TNBC, the results of which are yet to be published.

### 4.6. Proteolysis-Targeting Chimeras

Proteolysis-targeting chimeras (PROTACs), discovered in 2001 [70], are heterobifunctional molecules able to bind to a specific target with high affinity and covalently link to an E3 protein ligase, leading to target degradation. A few PROTACs have been investigated in in vitro and in vivo models of TNBC, mostly in MDA-MB-231 cells and xenografts, successfully inhibiting tumor growth (Table 2). Despite their potency, PROTACs show some drawbacks, including high molecular weight, poor cell penetration, low potency, and difficult synthesis. Currently, there are no clinical trials investigating the activity of PROTACs in TNBC.

## 5. Challenges and Limitations

There is a pressing need to improve treatment options for TNBC patients in order to minimize the side effects of the treatments while maintaining their quality of life. In this sense, the main challenge to tackle is the high interpatient and intratumor heterogeneity of TNBC. Moreover, there exists a genetic and epigenetic evolution of TNBC tumors over time, with a relevant change when the formation of metastatic lesions occurs. TNBC tumors can genetically and epigenetically drift between different TNBC subtypes, each of them distinguished by the up- or downregulation of different genes and proteins belonging to different signaling pathways responsible for their behavior, e.g., drug response and different tumor microenvironment compositions. High-throughput drug screenings in patient-derived xenografts with preserved intertumor and intratumor heterogeneity can effectively determine drug responses in genetically different TNBC [96]. However, only a multi-institutional effort can cope with TNBC heterogeneity. This has to provide a well-defined platform of patient biospecimens (tumor tissue, cell-free DNA/RNA, etc.) extracted at different time-points along all TNBC stages matched with patient clinical data, which include treatment interventions, occurrence of relapse, metastasis, etc. Then, genetic and immune changes can be correlated with specific tumor events and ultimately predict treatment responses within the different TNBC subtypes. Nonetheless, it is extremely difficult to put into practice.

Another challenge that diminishes treatment options in TNBC patients is the lack of novel therapeutically actionable biomarkers. Such a problem is partially linked to the high heterogeneity of TNBC tumors, where targeting a specific genetic aberration is only useful for a small proportion of TNBC patients. A few global genetic shRNA or CRISPR screens have been performed to identify novel synthetic lethal and drug resistance genes in TNBC [97,98]. However, these discoveries, which originated in well-characterized commercial TNBC cell lines, have not been translated to a significant increase in the available therapies, probably due to failure in posterior validation processes.

Another challenge to deal with is the difficulty of performing a long-term follow-up in TNBC patients due to the aggressiveness of the disease and the low survival rate. This limits the tumor response and the benefit of a given therapy in patients, as well as the long-term biological and clinical information of the tumor. Thus, novel surrogates’ endpoints to predict the treatment response are highly warranted.

TNBC frequently develop resistance to chemotherapy, which can be caused by different factors, including an increased expression of ATP-binding cassette (ABC) transporters, the existence of cancer stem cells, and the activation of multiple signaling pathways, such as the transforming growth factor-β (TGF-β), Notch, Wnt/β-Catenin, Hedgehog, hypoxia, nuclear factor kappa-light-chain-enhancer of activated B cells (NF-κB), PI3K/AKT/mammalian target of rapamycin (mTOR) pathways, Janus Kinase (JAK)/signal transducer, and activator of transcription (STAT) pathways [99]. This limits treatment efficacy, leading to treatment failure. Overall, chemoresistance is the result of the activity of a complex intricate of signaling pathways and factors, which, in combination with tumor heterogeneity, makes it very difficult to address. Many efforts have been made to suppress such processes. However, only a few drugs have transited to the clinic with the typical concerns of toxicity, selectivity, and specificity. The search for novel biomarkers to allow for the selection of patients that would benefit the most from certain chemosensitization agents becomes essential. In addition, combination regimens seem to be a successful strategy to mitigate drug resistance in TNBC.

## 6. Conclusions and Future Prospects

TNBC is not a single disease, but a highly heterogenous group of breast cancers with diverse genetic and epigenetic alterations, which limits the success rate and treatment efficacy. The acquisition of further knowledge profiling the molecular and immune landscapes of TNBC subtypes in depth can help in the identification of novel biomarkers crucial for optimal patient selection and for the development of personalized treatment that will lead to the improvement of treatment outcomes.

An interesting approach for doing so is the utilization of genome-wide in vivo CRISPR screens. Such a technique has recently allowed, for instance, the identification of a new immunotherapy target, *Mga* [100], or a new palbociclib sensitizer, TGFβ3 [101]. Moreover, the deployment of the cutting-edge methods such as spatial transcriptomics or single-cell sequencing can follow the spatial and timely evolution of tumor cells and the cancer environment at a single-cell resolution over time. If performed during the course of a particular treatment, it can provide information about the key players at different timepoints of cancer progression that can aid in the identification of novel resistance mechanisms. It can also be used for a comprehensive classification of TNBC, allowing for better patient selection, which is especially relevant given the high heterogeneity of TNBC.

Apart from that, TNBC research may benefit from recent technological advances in computations and artificial intelligence (AI). Currently, the main AI applications in cancer research are assisting in obtaining TNBC diagnosis accurately, providing more comprehensive TNBC subtyping, guiding treatment more precisely, and predicting TNBC prognosis with higher accuracy [102]. However, AI has to overcome a few obstacles which hinder its clinical application in TNBC, such as lack of accessibility, low standardization and incomplete clinical data, high cost of development, high demand of professionals in the field, and poor stability of AI models. However, the more scientists use the newest sequencing methods and AI, the more affordable, trained, and reliable they will become. It is envisaged that the widespread development of AI, together with the ready accessibility to the latest omics by worldwide laboratories and a multi-institutional collaborative effort to share clinical and molecular data, will allow for more precise patient stratification and the identification of novel biomarkers of treatment response for TNBC. Altogether, the aforementioned facts will improve treatment effectiveness in what is considered the breast cancer subtype with the worst clinical outcome.

## Figures and Tables

**Table 1:** Ongoing recruiting clinical trials for advanced or metastatic TNBC.

Start	Phase	Estimated No. Patients	Disease	Drug Regimen	Primary Outcome	Secondary Outcome	NCT No.
2022	I/II	20	Advanced or mTNBC	PD1+ TIL infusion	AE, ORR	PFS, CBR, DoR, OS	NCT05451784
2024	II	53	AR-positive mTNBC	Abemaciclib + Bicalutamide	DCR	AE, HRQoL, ORR, DoR, PFS, OS	NCT06365788
2024	II	36	Locally advanced unresectable or mTNBC	Trilaciclib + Pembrolizumab, Gemcitabine, and Carboplatin	ORR	PFS, DoR, OS, AE	NCT06027268
2024	III	350	Locally recurrent inoperable or resistant mTNBC	FDA018 (ADC) vs. ICC (Eribulin, Capecitabine, Gemcitabine, or Vinorelbine)	PFS, OS	ORR, DoR, DCR, AE, ADA	NCT06519370
2023	III	192	Basal-like immune suppressed (BLIS) subtype of TNBC	VEGFR BP102 + nab-Paclitaxel with maintenance of VEGFR + Capecitabine	PFS	ORR, DoR, OS, DCR	NCT05806060
2024	II	52	Unresectable, locally advanced, recurrent, or mTNBC	BL-B01D1 (ADC) + PD-1 mAB	ORR, RP2D	PFS, DCR, DoR, TEAE	NCT06471205
2024	III	406	Unresectable locally advanced or mTNBC after taxane failure	BL-B01D1 (ADC) vs. ICC	PFS, OS	ORR, DCR, DoR, TEAE, ADA	NCT06382142
2023	III	223	Immunomodulatory locally advanced or mTNBC	Famitinib (TKI) + Camrelizumab (anti-PD-1) and TPC (Nab-Paclitaxel, Capecitabine/Eribulin/Carboplatin) or Camrelizumab + TPC	PFS	ORR, DoR, CBR, OS	NCT05760378
2022	II/III	450	Untreated and inoperable locally advanced or mTNBC	BO13 (mAB) + Nab-Paclitaxel	ORR (phase II) PFS (phase III)	DCR, DoR, TTR, OS, CP, TEAE	NCT05555706
2022	II	63	Germline or somatic mutated BRCA1/2, PALB2 or RAD51C/D advanced patients Patients with RAD51-foci low score without known or with negative germline or somatic mutation in BRCA1, BRCA2, PALB2, RAD51C, or RAD51D	Olaparib	ORR	PFS, AE	NCT05340413
2024	I	30	mTNBC or locally advanced inoperable TNBC	Pembrolizumab + Cryoablation vs. Pembrolizumab alone	Changes in CD4-PD1 levels	-	NCT06246968
2023	II	78	Advanced or mTNBC	Utidelone (microtubule stabilizing agent) + Tirelizumab + Bevacizumab	ORR	PFS, DCR, OS, AEs	NCT06125080
2024	III	360	Inoperable locally advanced/mTNBC	PM8002 (anti-PD-L1/anti-VEGF-A bispecific antibody) + Nab-Paclitaxel vs. Placebo + Nab-Paclitaxel	PFS, OS	ORR, DCR, DoR, AE, HRQol	NCT06419621
2024	II	90	Advanced or metastatic breast cancer (TNBC or HR+/ER+/HER2-)	PCS6422 (Eniluracil) + Capecitabine vs. Capecitabine alone	ORR, AEs	DCR, DoR, TTR, PFS	NCT06568692
2018	III	657	Locally advanced or mTNBC	Nanosomal Docetaxel Lipid Suspension vs. Taxotere	ORR	PFS, OS, AE	NCT03671044
2023	II	46	Unresectable locally advanced or metastatic immunomodulatory TNBC	Camrelizumab + Famitinib with/without nab-Paclitaxel	PFS	ORR	NCT05670925
2023	III	203	LAR subtype with PI3K/AKT/mTOR mutation of locally recurrent inoperable or mTNBC	Everolimus + ICC (nab-Paclitaxel, Capecitabine, Eribulin, Carboplatin, Vinorelbine or Utidelone) vs. ICC alone	PFS	ORR, DoR, DCR, OS, AE, PRO	NCT05954442
2022	III	540	Previously untreated, locally advanced, inoperable or mTNBC with PD-L1 negative	Sacituzumab govitecan vs. TPC (Paclitaxel, nab-Paclitaxel, Gemcitabine + Carboplatin)	PFS	OS, ORR, DoR, TTR, TEAE	NCT05382299
2024	I/II	85	Advanced or metastatic refractory breast cancer	TIL therapy + IO (Cyclophosphamide, Fludarabine, IL-2, Pembrolizumab)	AE, ORR	DCR, DoR, PFS, OS, HRQol	NCT06532812
2023	II	36	HER2- metastatic or locally advanced metaplastic breast cancer (MpBC)	L-NMMA (iNOS inhibitor) and Nab-Paclitaxel combined with Alpelisib	RPD2, ORR	PFS, OS, PIK3CA status	NCT05660083
2024	II	44	Pretreated, locally advanced, or mTNBC	PLX038 (PEGylated prodrug of SN-38)	Best Tumor response (PR or CR)	TTR, SAEs, DoR, PFS, OS, AE, PK, PD	NCT06162351
2022	II	175	Unresectable locally advanced, recurrent, or metastatic HER2-negative breast cancer with no prior systemic therapy	Sacituzumab tirumotecan (SKB264) with/without KL-A167 (PD-L1 monoclonal Ab)	AEs, ORR	PFS, DoR, DCR, PK	NCT05445908
2017	I	70	Relapsed/refractory locally advanced BC or mTNBC	OTS167PO (MELK inhibitor)	MTD	-	NCT02926690
2022	I	26	Locally advanced or mTNBC or another solid tumor	BL-M02D1 (TROP2 ADC)	DLT, MTD, RP2D	TEAE, Cmax, Tmax, CL, ORR, DCR, DoR, PFS, ADA	NCT05339685
2023	II	160	Previously untreated, locally advanced, unresectable, or metastatic (stage IV) PD-L1 positive TNBC	Tobemstomig (bispecific Ab antiPD-1/anti-LAG3) + Nab Paclitaxel vs. Pembrolizumab + Nab-Paclitaxel	PFS	ORR, DoR, OS, ADA	NCT05852691
2016	I/II	229	Advanced or metastatic solid tumors (included TNBC)	LY2880070 (Chk1 inhibitor) alone and in combination with Gemcitabine	Maximum Tolerated Dose	DLT, Cmax, Tmax, ORR, PFS, OS	NCT02632448
2024	I	80	Unresectable, locally advanced, or metastatic solid tumor (included TNBC)	AGX101 (ADC therapy)	MTD, DLT, AE	PK, ADA, ORR, DCR, PFS, OS	NCT06440005
2021	I/IIa	48	Metastatic or advanced solid tumors (included TNBC)	BT-001 (oncolytic vaccinia virus) alone or in combination with Pembrolizumab	AE, iORR, iDCR	AE; DCR, PFS, DoR, OS	NCT04725331
2023	I	130	Locally advanced or metastatic solid tumor (included TNBC)	JANX008 (EGFR—TRACTr)	DLT, AE/SAE	Cmax, ADA, ORR, DoR, PFS	NCT05783622
2023	I	77	Metastatic solid tumor	PLN-101095 (specific integrin inhibitor) alone or in combination with Pembrolizumab	DLT	Cmax, PK, Tmax, DCR, ORR	NCT06270706
2022	I/II	354	Locally advanced, unresectable, or metastatic solid tumor (included TNBC)	PRO1184 (folate receptor α—ADC) in monotherapy or in combination with Carboplatin, Bevacizumab, or Pembrolizumab	TEAE, DLT, ORR,	DCR, PFS, OS, Cmax, Tmax	NCT05579366
2022	I/II	657	Different solid tumor, including TNBC with progression on or after treatment with at least one line of systemic CT in the advanced setting	NUV-868 (BD2-selective BET inhibitor) as monotherapy or with Olaparib or Enzalutamide	RPD2, DLT, PK, PFS, ORR	-	NCT05252390
2024	I	220	Advanced or metastatic solid tumor, expressing Nectin 4	LY4052031 (ADC targeting Nectin-4)	DLT, ORR, RP2D	PK, DoR, TTR, PFS, OS, DCR	NCT06465069
2024	I	280	Advanced or metastatic solid tumors known to express Nectin 4	LY4101174 (ADC targeting Nectin-4)	DLT, ORR, RP2D	PK, DoR, TTR, PFS, DCR, OS	NCT06238479
2023	I	48	Advanced or metastatic epithelial tumors including TNBC	MT-302 (TROP2-targeting mRNA-based CAR therapy)	AE, MTD, DLT, RP2D	PK	NCT05969041
2022	I/II	60	Locally advanced or metastatic TNBC without previous systematic treatment.	PM8002 (anti-PD-L1/anti-VEGF-A bispecific antibody) + Nab-Paclitaxel	ORR, TRAE	DCR, DoR, PFS, OS	NCT05918133
2020	I/II	116	Locally advanced or metastatic cancer, including TNBC	OC-001 (CD-137 mAB) as monotherapy or combined with Avelumab (anti-PD-1/PD-L1 Ab)	DLT, SAE, TEAE	Cmax, Chin, ORR, DoR, PFS, TTR, DCR, OS	NCT04260802
2021	I	345	Advanced malignancies include TNBC	NX-1607 (inhibitor of CBL-B) alone or in combination with Paclitaxel	TEAE, SAE, DLT, ORR	PK, DoR, DCR, PFS, OS, PD	NCT05107674
2022	III	646	Locally advanced or metastatic cancer in response to 6 months of standard immunotherapy (IO), including TNBC	Reduced dose intensity of IO vs. standard IO	PFS	ORR, OS, DoR, QL	NCT05078047
2022	I	100	Patients with ROR1+ relapsed or refractory TNBC and other cancers	LYL797 (ROR1-targeted CAR T-cell therapy)	DLT, TEAE, RP2D	ORR, DoR, PFS, OS, Cmax, Tmax	NCT05274451
2018	I/II	747	Advanced or metastatic solid tumors, including TNBC	Regorafenib + Avelumab (anti PD-L1 Ab)	RP2D, OR	MTD, DLT, PFS, OS, PK	NCT03475953
2021	I/II	115	Patients with advanced solid tumors	MDNA11 (IL-2 Superkine) alone or in combination with Pembrolizumab	TEAE, TRAE, DLT	Cmax, Tmax, ADA, ORR, DCR, PFS, TIL levels	NCT05086692

Abbreviations: ADA, antidrug antibody; ADC, antibody–drug conjugate; AE, adverse event; CBR, clinical benefit rate; Chk1, checkpoint kinase 1; CL, clearance; CP, drug plasma concentration; CR, complete response; DCR, disease control rate; DLT, dose-limiting toxicity; DoR, duration of response; HRQol, health-related quality of life; ICC, investigator’s choice of chemotherapy; iDCR, immune disease control rate; IL-2, interleukin 2; iNOS, inducible nitric oxidase synthase; IO, immunotherapy; iORR, immune overall response rate; LAR, luminal androgen receptor; mAB, monoclonal antibody; MTD, maximum tolerated dose; mTNBC, metastatic triple-negative breast cancer; ORR, objective/overall response rate; OS, overall survival; PD, pharmacodynamic; PD1, programmed cell death protein 1; PD-L1, programmed-death ligand 1; PFS, progression-free survival; PK, pharmacokinetic; PR, partial response; PRO, patient-reported outcome; QL, quality of life; ROR1, receptor tyrosine kinase-like orphan receptor 1; RP2D, recommended Phase II Dose; SAE, serious adverse events; TEAE, treatment-emergent adverse events; TIL, tumor-infiltrating lymphocyte; TKI, tyrosine kinase inhibitor; TPC, treatment of physician’s choice; TRAE, treatment-related adverse effect; TROP2, Trophoblast cell surface antigen 2; TTR, time to response; Tmax, time to maximum serum concentration; VEGF, vascular endothelial growth factor; VEGFR, vascular endothelial growth factor receptor; vs., versus.

**Table 2:** PROTACs studied in vitro and in vivo in TNBC.

PROTAC’s Name/s	Target	In Vitro	In Vivo	E3 Ligase	Ref.
Compound **29**	CDK9	MDA-MB-231 cells	MDA-MB-231 xenograft	CRBN	[70]
ZLC491	CDK12 and CDK13	MDA-MB-231 cells	MDA-MB-231 xenograft	CRBN	[71]
Compound **19s**	PAK1	MDA-MB-231 cells	MDA-MB-231 xenograft	CRBN and VHL	[72]
CEP1347-VHL-02	MLK3	MDA-MB-468 and HCC1806 cells	-	VHL	[73]
ARV-825	BRD4	MDA-MB-231 cells	MDA-MB-231 xenograft	CRBN	[74]
ARV-771 and MZ1	BET	MDA-MB-231 and MDA-MB-468 cells	-	VHL	[75]
Ganoderic acid A (GAA)	MDM2	MDA-MB-231 cells	MDA-MB-231 xenograft in zebrafish	VHL	[76]
MS8847	EZH2	MDA-MB-468 and BT549 cells, and BT549 3D cultures	-	VHL	[77]
US-10113	S100A4	MDA-MB-231 and 4T1 cells	4T1 allograft	CRBN	[78]
PU7-1	USP7-1	MDA-MB-468 and BT549 cells	MDA-MB-468 xenograft	CRBN and VHL	[79]
A4	CDK4/6	MDA-MB-231 cells	MDA-MB-231 xenograft	DCAF16	[80]
CT-4	HDAC8	MDA-MB-231 cells	-	CRBN	[81]
TEP	MYC	MDA-MB-468, Hs578T cells and 4T1	4T1 allograft	Pomalidomide	[82]
MZ1 and ARV-825	BRD2 and BRD4	MDA-MB-231 and BT549 cells	MDA-MB-231 xenograft	CRBN and VHL	[83]
MS83	BRD3 and BRD4	MDA-MB-231 and MDA-MB-468 cells	-	KEAP1	[84]
C8	PARP2	MDA-MB-231 cells	MDA-MB-231 xenograft	DCAF16	[85]
LB23	PARP1	MDA-MB-231 cells	-	CRBN	[86]
NN3	PARP1	MDA-MB-231 cells	MDA-MB-231 xenograft	MDM2	[87]
PP-C8	CDK12-Cyclin K complex	MDA-MB-231 cells	-	CRBN	[88]
IY-IY-pom	TrkC	Hs578T	-	CRBN	[89]
Compound **6n**	AXL kinase	MDA-MB-231 and MDA-MB-468 cells, and TNBC patient organoids	MDA-MB-231 xenograft	CRBN	[90]
UI3i	EZH2	MDA-MB-231 and MDA-MB-468	-	CRBN	[91]
MS8815	EZH2	MDA-MB-453, BT549 and patient-derived TNBC cells	-	VHL	[92]
AR-PROTAC	AR	BT549	BT549 xenograft	VHL	[93]
dTRIM24	TRIM24	Metaplastic and non-metaplastic TNBC tumorspheres	-	VHL	[94]
Compound **45**	CDK9	MDA-MB-231 cells	MDA-MB-231 xenograft	CRBN	[95]

Abbreviations: AR, androgen receptor; BET, bromodomain and extra terminal; BRD, bromodomain; CDK, cyclin-dependent kinase; CRBN, Cereblon; DCAF16, DDB1- and CUL4-associated factor 16; EZH2, Enhancer of Zeste Homolog 2; KEAP1, Kelch-like ECH-associated protein 1; HDAC, histone deacetylase; MDM2, mouse double minute 2 homolog; MLK3, mixed-lineage protein kinase 3; PAK1, P21 (RAC)1 activated kinase 1; PARP, Polyadenosine 5′-diphosphoribose polymerase; TrkC, Tropomyosin receptor kinase C; USP7, Ubiquitin-specific-processing protease 7; VHL, Von Hippel–Lindau tumor suppressor.

## Data Availability

No new data were created.
